# Association between Social Integration and Health among Internal Migrants in ZhongShan, China

**DOI:** 10.1371/journal.pone.0148397

**Published:** 2016-02-10

**Authors:** Yanwei Lin, Qi Zhang, Wen Chen, Jingrong Shi, Siqi Han, Xiaolei Song, Yong Xu, Li Ling

**Affiliations:** 1 Faculty of Medical Statistics and Epidemiology, School of Public Health, Sun Yat-sen University, Guangzhou, China; 2 Center for Migrant Health Policy, Sun Yat-sen University, Guangzhou, China; 3 School of Public Health, Baotou Medical College, Baotou, China; 4 School of Community and Environmental Health, Old Dominion University, Norfolk, Virginia, United States of America; School of Public Health, Fudan University, CHINA

## Abstract

Internal migrants are the individuals who migrate between regions in one country. The number of internal migrants were estimated at 245 million in China in 2013. Results were inconsistent in the literature about the relationship between their health statuses and social integration. The main difference exists on how to measure the social integration and whether health statuses of internal migrants improve with years of residence. To complement the existing literature, this study measured social integration more comprehensively and estimated the internal migrants’ health statuses with varying years of residence, and explored the associations between the migrants’ social integration and health. We used the data from 2014 Internal Migrant Dynamic Monitoring Survey of Health and Family Planning in ZhongShan, China. Health status was measured from four aspects: self-reported health, subjective well-being, perception of stress, mental health. We measured social integration through four dimensions: economy, social communication, acculturation, and self-identity. The analyses used multiple linear regressions to examine the associations between self-reported health, subjective well-being, and perception of stress, mental health and social integration. The analytical sample included 1,999 households of the internal migrants and 1,997 local registered households, who were permanent residents in ZhongShan. Among the internal migrants, Adults in the labor force, who were aged 25 to 44 years old, accounted for 91.2% of the internal migrant population, while 74.6% of the registered population were in that age group. Median residential time among migrants was 2.8 (1.3–6.2) years, and 20.2% of them were migrating in the same Guangdong province. Except for mental health, other health statuses among migrants had significant differences compared with local registered population, e.g. self-reported health was better, but subjective well-being was worse. However, these health measurements were improved with more years of residence. Moreover, our results show that two aspects of social integration, economic integration and self-identity, were significantly associated with health status. Subjective feeling of relative social status levels were more associated with health, which prompted the attention to social fairness and the creation of a fair and respectful culture. More interventions could be experimented, such as encouraging internal migrants to participate in community activities more actively, educating local registered residents to treat internal migrants more equally, and developing self-identity among internal migrants. Better social, economic, and cultural environment can benefit internal migrants’ health statuses.

## Introduction

Along with the economic development and the urbanization, China emerges with a large number of internal migrating population. According to Report on China’s Migrant Population Development of 2014, an estimated 245 million internal migrants who were originally from the largely poor and rural areas in the western and central inland provinces and migrated to the eastern developed regions, such as Beijing, Shanghai, Guangdong for better job opportunities and income at end of 2013. In our study, we adopted the definition of internal migrants in the National Internal Migrant Dynamic Monitoring Survey, in which internal migrants were the individuals who did not have “Hukou” (permanent registered resident certificate) and should be living over a month in the residence. Due to long-standing household registration (“Hukou”) policy, with its dual governance system between rural and urban areas, internal migrants do not have the same rights and benefits as local registered population in a variety of areas, such as employment, education, housing, health care, and social services. How the migration process affects internal migrants’ health statuses continuously attract researchers’ interests. Like international migrants, internal migrants experience similar acculturation since China has significant disparities in culture, economic development, and social environment across regions [1, 2].

A growing number of studies suggest that migration has an impact on both physical health and mental health, while most of the research focused on the migrant population in the U.S. and other developed countries [3–5], and some of studies focused on Chinese internal migrants [6, 7]. The existing literature provides the evidence to support the “healthy migrant paradox”, i.e. immigrants have physical health advantages over the native-born at the initial stage of the immigration. However, the immigrant health advantage diminishes significantly with increasing resident time in the host society [6, 8, 9]. Some studies documented the high level of psychological distress among immigrants. However, the mental outcomes of immigrants can differ over time of the residence. Therefore, further evidence is needed to clarify the relationship between migration and mental health as well [10].

Social integration theory were developed to understand and explain the achievements of immigrants’ behavior, adaptation, cultural fusion, the acculturation process and outcome of identity, although most applications were in western society [11]. Social integration is a multi-dimensional concept without a clear and unified definition among researchers across disciplines [12]. Consequently, measure of social integration is not definitive and a typical operational definition was based on a scale with individual summary scores across types of social roles or social networks [13]. In this study, we adopted the theoretical framework of social integration proposed in Yang (2010)[14], which measured the social integration with four dimensions- economic status, social communication, acculturation, and self-identity.

The relationship between social integration and health could be complicated among immigrants. For example, in some studies on immigrants in the United States or other developed countries, acculturation process is hypothesized to have stress on immigrants which could negatively affect the immigrants’ mental health status [10, 15]. However, on the other perspective, the initial acculturation stresses may be reduced with immigrants becoming more familiar with the society, which could reduce the stress over time [16]. It is also possible that highly acculturated individuals are sensitive to negative stressors, such as invisible discrimination, which could increase the stress level with longer time of residency [17]. Gender, socio-economic status, family, and community support were important factors that may determine the direction of changes in health statuses in the process of acculturation [18–20]. For example, immigrants living in communities with stronger social support had better mental health and occupational rehabilitation [21, 22].

Social integration process also means the improvement of social network, less social strains, and with more social support, which are all possibly linked with better health statuses [23]. Social network can be measured quantitatively (e.g. social network size) or qualitatively (e.g., perception of loneliness). Worse social network could be associated with poor health statuses prospectively, e.g. the increased risk for morbidity and mortality [24]. Social strains were strongly and positively related to coronary artery risk [25]. Social integration may also reduce health disparities, which could be negatively associated with immigrants’ health status, such as hospitalization rates for stroke, cervical cancer, and appendectomy [26–30].

While traditional research focused on direct measurement of internal migrants’ health statuses, such as reproductive health, infectious diseases, mental health, health behaviors, or occupational health risks [31–34], this paper explored the potential factors associated with internal migrants’ health statues, such as social integration. Our objective is to complement the existing literature by providing further insights into the social factors which might influence the health statuses of Chinese internal migrants. Our results may motivate the policy makers to adopt right approach to improve internal migrants’ social integration.

## Materials and Method

### Study Site and Data Collection

We selected ZhongShan, a city in Guangdong Province in China as the study site since it is located in the middle of the Pearl River Delta, one of the two major internal migrant resident areas in China. The local economy relies on the factories and the service industry, which attracts large number of migrant workers from other regions in Guangdong or less developed provinces in China. In 2014, ZhongShan has approximately 1.65 million internal migrants, almost 50% of the total population [35].

The data came from the Internal Migrant Dynamic Monitoring Survey conducted by the National Population and Family Planning Commission in 2014. We used the data from ZhongShan, one of the sampling cities in the survey. This survey included standardized measurement of social integration and health statuses among internal migrants. Interviewers received standardized training by local health department staff, and quality control was implemented in data collection. The stratified, multi-stage sampling was adopted based on Probability Proportionate to Size Sampling method (PPS). The basic sampling framework for internal migrants were all migrants who were reported by each village or neighborhood in Zhongshan, while the sampling framework for registered residents was based on actual census. Multilevel random selection was applied. Township were randomly selected and followed by village or neighborhoods. In each village or neighborhood, 20 individual subjects were selected. The targeted population included two groups of residents. First was internal migrants who did not have “Hukou” (registered resident certificate) in ZhongShan and should be living over a month in ZhongShan. The comparison group was registered population who had “HuKou” in ZhongShan. All sampled respondents were between 18 and 59 years of age.

### Measurement

We listed the definitions of the variables in Appendix (S1 File) and described them as follows:

#### Health

In this study, we measured internal migrant’s health status from four aspects: self-reported health, subjective well-being, perception of stress and mental health. Self-reported health is one of the internationally accepted health indicators reflecting a person’s perception of overall health, which is a strong, independent, and reliable predictor of morbidity [36]. The subjective well-being measures a person’s self-rated assessment of quality of life [37]. Perception of stress is a subjective evaluation of stress that plays an important role in emotional and physiological responses [38]. Mental health indicates the overall psychological well-being, which accounts for an important dimension of health measurement for internal migrants [39, 40]

Some studies noted that immigrants had higher distress than the native population at the initial stage of their immigration process [6, 41]. However, Hener et al. (1997) suggested that there was less depression amongst migrants 6 months after migration. The evidence in the literature is inconsistent about how the distress level changes among immigrants after 1 year of migration [42, 43]. Pernice et al. (2009) found that immigrants presented poor mental health in the first 2 years post-migration. Lerner et al. (2005) discovered that objective adaptation among immigrants had occurred to a greater degree over 5 years [44]. Aroian and Norris (2002) stressed that depression in immigrants should be anticipated in the first 5 to 7 years of resettlement [45]. Studies had emphasized the effect of duration of stay on general health among immigrants seems to be more favorable than that of the host population in the first 10 years post-migration [46]. In light of the above researches, we divided years of residence into 8 levels: 1 as 1–6 months, 2 as 6–12 months, 3 as 1–2 years, 4 as 2–3 years, 5 as 3–4 years, 6 as 4–5 years, 7 as 5–10 years, 8 as over 10 years. We compared the health statuses between internal migrants and registered population at every level.

We used different scales to measure self-reported health, subjective well-being, perception of stress and mental health. Self- reported health was measured by general health (GH) dimension of SF-36, which is widely used to measure quality of life assessment tool in the world [47]. SF-36 evaluates health related quality of life from 8 aspects—physical functioning, physical role function, bodily pain, general health, vitality, social role functioning, emotional role function, and mental health. In our study we chose its general health dimension to appraise self-reported health, which was measured with five items and each item used a five point scale. The Chinese version of SF36 was tested with a Cronbach’s coefficient for GH dimension at 0.72 [48].

Satisfaction with Life Scale (SWLS) was used to measure subjective well-being. The SWLS assesses the global life satisfaction and includes 5-items on a seven point scale from 1 (disagree strongly) to 7 (agree strongly)[49]. The Cronbach’s coefficient for SWLS in Chinese is 0.732 [2]

Perception of stress was measured by four items: 1) the subject was unable to control the important things in the subject’s life; 2) the subject was confident about his or her ability to handle personal problems; 3) the subjects felt that things were going his or her way; 4) the subject felt that difficulties were piling up so high that he could not overcome them. These items were adopted from the perceived stress scale (PSS-10)[50] and could measure both negative stress and positive stress. Factor loadings of question 1 and 4 are 0.65 in negative stress dimension of PSS scale, and factor loadings of question 2 and 3 are 0.72 and 0.70 in positive stress dimension respectively [51]. Higher composite scores indicate less perceived stress.

The K6 scale of psychological well-being was used to evaluate mental health [29]. It was developed to assess the distribution of general distress and to screen for cases of mental illness in general population and has been used in large-scale surveys such as the National Health Interview Survey (NHANES) in the U.S. and all the national surveys in the World Health Organization’s World Mental Health (WMH) Initiative [52]. The scale consists of six items: the respondents were asked how often they felt nervous, hopeless, anxious, depressed, and worthless or that everything had been an effort during the past 30 days. Each question can be answered from 1–5 in an ordinal scale from 1 (all of the time) and 5 (none of the time). The internal consistency reliability is acceptable (0.80)[26]. Higher values on the scale represent better mental health.

#### Social Integration

As mentioned above, we measured social integration through four dimensions: economic status, social communication, acculturation, and self-identity. The variables selected to measure social integration referred to the indicator system proposed by Yang (2010) [14] and Zhou (2012)[53]. Since self-perception of the relative socioeconomic status may influence the economic integration [54], we added the subjective economic indicators (income, occupation position and level of respect compared with other people in the society, such as family members, friends and colleagues at the current residence or at their hometown). Employment, labor contract, household income and daily working time represented the stability of employment, income level and the labor intensity respectively.

We used seven indicators to appraise internal migrants’ economic status: employment, labor contract, household income, daily working time, subjective social status and level of respect (1 as the least to 10 as the best) compared with other people in the society, such as family members, friends and colleagues at the current residence or at their hometown.

Social communication was measured with three questions:1) How many organizations the subjects joined, such as labor union, volunteer association, the Chinese Communist Party group of migrants/local residents, alumni association, chamber of commerce of hometown, association of migrants from the same hometown and other organizations; 2) Has the subject participated in the following activities, such as community sports, social public welfare activities, election campaign, awards events, the home owners' committee, management activities of residents' committees and other activities; 3) What types of the subject’s neighbors are, such as migrants, native born, or unknown. Quantitative and categorical answers to these questions were summarized as the measures of social communication.

Acculturation was measured by views about social norms adopted and primary language used. Those views include 7 items about social norms: 1) The customs at hometown (such as the customs of marriage, funerals) is more important to the subject; 2) Working in the current place is more important to me than living at hometown; 3) The subject’s child should learn to speak hometown dialect; 4) Maintaining the hometown's lifestyle, such as eating habits, is important; 5) There is a big difference on health habits between the subject and local residents; 6) There is a big difference on clothing between the subject and local residents; 6) There is a big difference on education or retirement style between the subject and local residents; 7) The subject’s opinions of some social problems are very different from the local residents’. Respondents were asked to report their agreements with these views based on five point scale (strongly agree, agree, neither agree or not, disagree, strongly disagree). In addition, ZhongShan dialect is Cantonese, so respondent was asked about how familiar with Cantonese.

Self-identity was measured by integration will and subjective identity. Integration will consists of 13 questions, such as “I would like to live together with locals in a block (community)”, “I would like to be a colleague with locals” and “I would like to be neighbor with locals”. Respondents were asked about the level of agreement with these statements based on four point scales (1 as disagree completely and 4 as agree completely) and the higher score means better integration will. The subjective identity was measured by a binary variable (1 as thought of himself/herself as a local resident, 2 as not)

### Statistical Methods

Descriptive analyses were performed to present demographic information, health (self-perceived health, subjective well-being, perception of stress and mental health), and internal migrants’ social integration. Student’s T-test was used to test the difference in health between internal migrants and registered population, and Dunett t (2-side) was used to comparing the health statuses within groups. Multi-variate linear regressions were used to explore the associations between health statuses and social integration. Gender, age, education and marital status were controlled after the stepwise regression methods were applied for model selection. We used SPSS20.0 to conduct the statistical analyses [55].

### Ethics

Written consent was sought from eligible participants, and all of participants were adults (over 18 years old). About minors/children information, we obtained informed consent from their guardians, and consent on behalf of the children enrolled was written. The study was approved by the Ethics Committee of the School of Public health, Sun Yat-sen University, China.

## Results

### Demographic characteristics

The study sample included 1,999 internal migrants and 1,997 registered residents in ZhongShan, China. 51.6% of internal migrants were men and 48.4% were women. Adults aged 25 to 44 years old accounted for 91.2% of the internal migrant population. But 74.6% of the registered population were in that age group. Lower level of education was observed in internal migrants: 93.6% of internal migrants and 61.8% of registered population received education lower than junior college respectively. More internal migrants were married (89.3% vs. 81.5% in internal migrants and registered population respectively). Details was showed in Table 1.

**Table 1:** Respondent’s Socio-demographic Characteristics in ZhongShan in 2014.

variable	Internal Migrants (%)	Registered Population (%)	Variable	Internal Migrants (%)	Registered Population (%)
**gender**			education***		
man	1031(51.6)	1082(54.2)	Never be educated	9(0.5) -	14(0.7)-
female	968(48.4)	915(45.8)	Primary school	213(10.7)-	185(9.3)-
ratio of Male and female	1.07	1.14	Middle school	1161(58.1) -	563(28.2)-
age***(years old)			High school/technical secondary school	486(24.3)	472(23.6)
18~	243(12.2)	253(12.7)	Junior College	99(5.0)	333(16.7)
25~	878(43.9)	660(33.0)	Undergraduate	29(1.5)	222(11.1)
35~	702(35.1)	578(28.9)	postgraduate	2(0.1)	8(0.4)
45~	176(8.2)	386(19.3)	marital status***		
55~59	3(0.2)	120(6.0)	single	201(10.1)	317(15.9)
total	1999(100)	1997(100)	marriage	1785(89.3)	1662(81.5)
			divorce	9(0.5)	28(1.4)
			widowed	4(0.27)	26(1.3)

*** p<0.001

### Migrating Characteristics

79.8% of internal migrants were from other provinces in addition to Guangdong province, i.e., out-of-province migrants and 20.2% of internal migrants were from Guangdong province, i.e., within-province migrants. The top three provinces of migrant sources except Guangdong were Guangxi, Hunan, Sichuan, which accounted for 20.9%, 14.4% and 20.9% of internal migrants respectively. Median of migrating time was 2.8 years, Interquartile range(IQR) was 1.17–6.25years, and migrants with shorter than 3 years of residences in ZhongShan accounted for more than half of the population, and 13.2% of migrants with residence time over 10 years. The main reason of migrating into ZhongShan were working or engaging in trade (95.9%). Details was showed in Table 2.

**Table 2:** Respondent’s Years of Residences and Migrating Reasons in ZhongShan in 2014.

Years of residence	Population (%)	Migrating reason	Population (%)
1~6 months	232(11.6)	working or engaging in trade	1,917(95.9)
6 months~1 year	211(10.6)	Family reasons	64(3.2)
1 year~	379(19.0)	Marriage	1(0.1)
2years~	239(12.0)	House relocation	1(0.1)
3years~	153(7.7)	Visit relatives	10(0.5)
4years~	140(7.0)	Other reason	5(0.3)
5years~	381(19.1)	Total	1,999(100)
≥10years	264(13.2)		
total	1,999(100)		

### Social Integration

Table 3 presented the statistics of social integration among internal migrants. In the aspect of employment rates, internal migrants in ZhongShan had a higher rate than registered populations (89.6% vs. 83.6%), but the employment rate among internal migrants in ZhongShan was lower than the national average employment rate of all internal migrants in China in 2012 (97%) [56]. A broader measure of employment is the number of people with a labor contract signed. That rate was lower in internal migrants than in registered populations (74.2% vs. 89.8%), i.e. many internal migrants worked without a labor contract. The internal migrants worked more intensely than registered populations. 50.7% of internal migrants worked more than eight hours of working time compared with 21.5% of registered populations. However, although internal migrants worked for longer hours, family income was lower compared with registered population. The interquartile range of 25% to 75% of internal migrants’ monthly income was 4,000–7,000 RMB (or 643 USD to 1,125 USD) and registered populations’ range was between 5,000 and 8,000RMB(or 804 USD and 1,286 USD. The internal migrants also ranked their income or occupation positions with the whole society, with close people (e.g. friends or colleagues) in ZhongShan, or with close people in their hometown (0 was the lowest, 10 was the highest). The mean score of the position compared with the whole society was 4.8, the mean compared with friends or colleagues in ZhongShan was 5.51 and the score compared with friends or colleagues in their hometown was 5.69.

**Table 3:** Internal Migrants’ Social Integration in 2014 in ZhongShan.

	$\bar{\text{x}}$ ±s or population (%)
**Economy**	
Employment	
Yes	1792(89.6)
No	207(10.4)
Labor contract	
Unfixed term	186(14.1)
fixed term	780(59.3)
one-time task or a probation period	10(0.8)
unsign labor contract	257(19.5)
unknown	81(6.2)
else	2(0.2)
Household income*	4000–7000
Work time every day	8.83±2.87
Income, occupation position compared with the people of the whole society(1–10)	4.80±1.68
Income, occupation position compared with the relatives, friends and colleagues of the current residence(1–10)	5.51±1.67
Income, occupation position compared with friends and colleagues of their hometown(1–10)	5.69±1.67
Degree of respect compared with whole society(1–10)	5.35±1.77
Degree of respect compared with relatives, friends and colleagues of the current residence(1–10)	6.1±1.62
Degree of respect compared with friends and colleagues of their hometown(1–10)	6.34±1.67
**Social communication**	
Number of organizations participated (0–8)	0.16±0.52
Number of activities attended(0–7)	0.33±0.71
Type of neighbors	
Outsiders	1016(50.8)
The locals	207(10.4)
outsiders and locals	692(34.6)
Not sure	84(4.2)
**Acculturation**	
The consent of the views(8–40)	22.87±4.26
Familiarity with the local dialect	
understand and speak	402(20.1)
understand and speak some	473(23.7)
understand some only	770(38.5)
Don't understand	354(17.7)
**Self-identity**	
Integration will(13–52)	38.85±4.29
Think oneself native or not	
Yes	396(19.8)
No	1603(80.2)
Bring family members or not to the local in the next 1–3 years	
All of family members at here	577(28.9)
Yes	210(10.5)
Yes, but some	531(26.6)
No	556(27.8)
Not sure	125(6.3)

* interquartile

Internal migrants hardly participated in any social organizations and activities (both of the mean scores were lower than 0.5 out 10 point scale). More than 50% of neighbor types were migrants. With regards to the acculturation to local customs, the score was 22.87 out of 40 point scale, which suggests that internal migrants on average was neutral between hometown customs and local customs. The proportion of internal migrants who can understand and speak Cantonese was 20.1%, while 80.2% of internal migrants thought of themselves as outsiders. This may suggest the language skill could be an important indicator for self-identity. But the will to integrate was strong with the mean score of 38.85 out of 52 point scale. 28.9% of internal migrants had already all their family members in town, while another 37.1% of internal migrants planned to bring at least some family members to town within next three years.

### Health Statuses

The health status of registered population was the benchmark. We found significant differences between internal migrants and registered population in the aspect of self-reported health, subjective well-being, perception of stress, but not mental health. The self-reported health of internal migrants was better than that of registered population. In terms of subjective well-being and perception of stress, internal migrants was worse than registered population. With longer years of residence, self-reported health of internal migrants was significantly better than that of the registered population. The relationship between residence years and the subjective well-being was not clear. The perception of stress was initially higher in internal migrants than in registered population. However, the level of perception of stress was almost monotonically decreasing with longer years of residence. Details was showed in Table 4.

**Table 4:** Respondent’s health situation in 2014 in ZhongShan ($\bar{\text{x}}$ ±s).

	Registered population	Internal migrants	Years of residence of internal migrants							
			Level1	Level2	Level3	Level4	Level5	Level6	Level7	Level8
Self-reported health	21.43±3.95	22.31±3.86***	21.41±3.56	21.82±3.57	22.10±3.57*	22.46±3.89**	22.10±4.36	22.37±3.93*	22.83±3.92*	22.98±4.09*
subjective well-being	22.03±6.23	21.53±5.86**	22.21±5.58	22.11±5.06	21.40±5.59	21.30±6.23	21.03±5.73	21.13±5.61	22.00±6.23	22.35±6.34
perception of stress	14.20±2.54	13.96±2.49**	13.56±2.34**	13.55±2.30**	13.65±2.11**	13.92±2.52	14.16±2.42	13.86±2.57	14.28±2.70	14.59±2.70
Mental health	26.36±3.16	26.43±3.07	26.17±3.32*	26.11±3.35	26.52±2.91	26.56±2.98	26.54±2.71	25.87±3.53	26.59±3.04	26.66±2.82

* p<0.05;

** p<0.01;

*** p<0.001

### Relationship between Health and Social Integration

The regression results indicated significant relationship between social integration and health statuses among internal migrant in ZhongShan, China (Table 5). In our models, stepwise selection method was used to select variables, and we referred to adjusted R^2^ to choose models. Demographic characteristics were significantly associated with self-reported health and mental health. Male migrants had worse self-reported health (P<0.001) and worse mental health (P<0.05). Interestingly, migrants with better education had worse self-reported health (P<0.05) and worse mental health (P<0.01). Years of residence was positively associated with self-reported health and perception of stress (P<0.001), i.e. with longer residence time, migrants had better self-reported health and experienced with lower level of stress at the same time. In economic integration aspects, the relative position in income or occupation in the whole society was significantly and positively related to self-reported health (P<0.001), subjective well-being (P<0.001), and mental health (P<0.01). Degree of respect received from others also had a positive association with health statuses. Working time was negatively associated with health statuses, especially subject well-being (P<0.01). Acculturation had no consistent and significant relationship with four health measures. However, self-identity was significantly associated with the health statuses. For example, stronger integration will was positively and significantly related to all four health statuses. Furthermore, identifying themselves more as locals or less as outsiders were positively related with health statuses. Internal migrants with more family members in town or planning to bring members in town were associated with better subjective well-being and mental health.

**Table 5:** Coefficients of Multi-variate Regressions of the Relationship between Social Integration and Health among Internal Migrants in ZhongShan, China.

	Self-reported health		Subjective well-being		Perception of stress		Mental health	
	B	SE/*t*	B	SE/*t*	B	SE	B	SE/*t*
**demographic characteristics**								
Gender	-0.826***	0.021	0.657*	0.285	-0.157	0.131	-0.349*	0.162
Age	-0.071***	0.015	0.042	0.021	0.005	0.010	-0.013	0.012
Education	-0.282*	0.125	-0.177	0.183	0.044	0.081	-0.349**	0.102
Marital status	-1.111	0.274	0.300	0.391	-0.33	0.178	0.231	0.222
Years of residence	0.271***	0.045	-0.002	*-0*.*066*	0.133***	0.029	0.042	*1*.*490*
**Economy**								
Labor contract	0.03	*1*.*116*	-0.299*	0.126	-0.028	*-1*.*019*	0.011	*0*.*385*
Household income	0.15	*0*.*566*	0.031	*1*.*226*	0.010	*0*.*380*	0.022	*0*.*838*
Work time every day	-0.031	*-1*.*153*	-0.206*	0.105	-0.009	*-0*.*317*	-0.038	*-1*.*400*
Income, occupation position compared with the people of the whole society	0.259***	0.066	0.857***	0.112	-0.015	*-0*.*462*	0.168**	*0*.*062*
Income, occupation position compared with the relatives, friends and colleagues of the current residence	-0.002	*-0*.*069*	0.254*	0.120	-0.004	*-0*.*117*	0.010	*0*.*304*
Income, occupation position compared with friends and colleagues of their hometown	0.060	*1*.*762*	-0.014	*-0*.*328*	-0.010	*-0*.*317*	0.015	*0*.*484*
Degree of respect compared with whole society	-0.013	*-0*.*366*	0.069	*1*.*880*	0.164**	0.049	0.222***	0.057
Degree of respect compared with relatives, friends and colleagues of the current residence	0.002	*0*.*054*	0.503**	0.156	0.174**	0.053	0.059	*1*.*677*
Degree of respect compared with friends and colleagues of their hometown	0.339***	*0*.*064*	0.355*	0.148	0.030	*0*.*678*	0.013	*0*.*415*
**Social communication**								
Number of organizations participated	0.028	*0*.*984*	0.039	*1*.*508*	0.031	*1*.*186*	-0.004	*-0*.*131*
Number of activities attended	0.288*	0.132	0.013	*0*.*505*	-0.035	*-1*.*306*	-0.525***	0.107
Type of neighbors	-0.033	*-1*.*249*	-0.015	*-0*.*589*	-0.135*	0.064	0.026	*0*.*977*
**Acculturation**								
The consent of the views	-0.022	*-0*.*832*	0.018	*0*.*701*	-0.003	*-0*.*127*	-0.013	*0*.*415*
Familiarity with the local dialect	-0.002	*-0*.*069*	-0.007	*-0*.*249*	-0.002	*-0*.*078*	-0.017	*-0*.*618*
**Self-identity**								
Integration will	0.083***	0.025	0.092*	0.036	0.050**	0.016	0.046*	0.020
Think oneself native or not	-0.550*	0.270	-1.101**	0.385	-0.557**	0.175	-0.006	*-0*.*230*
Bring family members or not to the local in the next 1–3 years	-0.040	*-1*.*469*	-0.395***	0.117	-0.036	*-1*.*287*	-0.171**	0.066
Constant	25.425	0.856	14.931***	2.494	10.647***	0.965	25.050***	1.106
Wald F Statistics	17.510***		21.028***		13.694***		11.525***	

B = b coefficient. SE = standard error.

* p<0.05;

** p<0.01;

*** p<0.001

## Discussion

As a representative city in the largest migrant-concentrated region in China, ZhongShan provided a good site to examine the relationship between social integration and health among internal migrants (62). Therefore, our results provide the initial evidence to understand how the complicated migration and acculturation process may be associated with internal migrants’ health statuses in different dimensions.

This study advances the measurement of social integration with more dimensions, while existing literature uses social interaction or cultural integration among internal migrants [57]. In the economic integration, internal migrants had better employment rates than local residents while the working time could be longer with lower income. The internal migrants were still socially isolated and rarely participated in any social organizations or activities, which could potentially hurt their physical or mental health [22]. This result has high policy relevance for local government to promote more social integration among internal migrants [21]. More incentives or promotions should be arranged for internal migrants to participate in these local social activities, such as community sports [58]. Moreover, for migrants with strong will to integrate, local government or communities should create the infrastructure or environment for them to bring their family members to town, so that their health could be improved with longer time of residence [59]. Since the self-identity was also protective to migrants’ health, the employers or the local communities can take more active steps to boost their self-identity as local residents [60, 61].

The healthy migrant paradox was observed among internal migrants in terms of self-reported health, which was consistent with previous research [6, 62]. However, our contributions to the literature were to find the healthy migrant effect can be enhanced with longer time of residence. One of the hypotheses to explain the results was the social integration did improve many aspects of health among internal migrants. Interesting, we did not observe significant disparity in mental health between internal migrants and local residents, which provides new evidence to support migrants’ mental health was not necessarily worse than registered population [1, 33, 63, 64]. Because of the complicated process of the migration and integration process into local society, different health measurements may have opposite relationship with the various factors of social integration, such as acculturation [9], A study showed stress of acculturation was related to sexual-risk behaviors and related mental health in 18-24-year-old internal migrants in Shanghai, China [65], but in our study acculturation was not associated with self-reported health, subjective well-being, perception of stress and mental health, and for self-reported health, the "salmon bias" (healthier individuals are more likely to migrate and to shift further away from home) was supported [62], which cautions the policy makers to implement the social integration tools carefully in migrant populations.

The following limitation was acknowledged to avoid over-interpretation of our results. As the first study to use the comprehensive measurement of social integration, our results were unable to be full compared with the evidence in the existing literature; meanwhile, alternative definition of resident vs. internal migrant is possible, e.g. 'born in Zhang Shan area' as a measure to differentiate locals vs migrants so our results could be biased more towards more recently migrated population. Moreover, the measure of social communication/participation could be less favorable towards internal migrants, i.e. more favorable to residents. Moreover, this is a cross-sectional study, so we can only examine the association between social integration and health, instead of the causation between these two measurements. Therefore, we propose more research, such as a cohort study, to understand the causation and direction of social integration and health, e.g. whether healthier migrants have stronger will to integrate into local society. ZhongShan is a mid-size city in China and may not represent the social, cultural, and economic environment in these metropolitan areas with much more migrant population, such as Beijing and Shanghai. The relationship between social integration and health could be more complicated than the relationship in ZhongShan. More carefully designed studies are needed in these large cities in China.

In summary, this study provides a more in-depth examination of health and social integration among internal migrants in China. We confirmed the positive association between social integration and health in a variety of dimensions which contributed the existing literature in the field [13, 22, 66]. The measurement of social integration in this study were more comprehensive and specific than previous studies [2]. Among all these social integration factors, relative socioeconomic position in the society and self-identifies were the most significant factors associated with internal migrants’ health status. The time analyses of these effects also provided a guidance to local government about the optimal time to enhance social integration to improve health among migrants, i.e. internal migrants within 2 years of residents might be the target group for intervention. The intervention could include but not limited to promote migrants’ participation to local organization and community events, boost self-identity among migrants, and create more favorable conditions for migrants’ family reunion. Due to the significant number of internal migrants in China, all these interventions can achieve a significant increase in welfare of one of the largest population group in China.

## Supporting Information

S1 File Appendix: Definition’s Specifications of the Selected Variables. (DOCX)
