# Supplementary material for: Association between Social Integration and Health among Internal Migrants in ZhongShan, China
Source: PLoS One. 2016 Feb 10;11(2):e0148397. doi: 10.1371/journal.pone.0148397 (PMC4749174; doi:10.1371/journal.pone.0148397)
Supplement: S1 File — (DOCX) [file pone.0148397.s001.docx]

S1File. Definition’s Specifications of the Selected Variables

| **dimensionality** | **variables** | **Definition** |
| --- | --- | --- |
| Health | Self-reported health | the subject’s perception of overall health |
|  | Subjective well-being | The subject’s self-rated assessment of their quality of life |
|  | Perception of stress | subjective evaluation of stress |
|  | Mental health | the overall psychological well-being |
| Economy | Employment | Employment status in the week before the survey |
|  | Household income | Total income of all family members in the local residence. |
|  | Daily working time | average daily number of working hours |
|  | Income and occupation position compared with the people of the city | Subjective social status and level of respect compared to the other people which was measured by marking a "social ladder" (1 as the bottom status to 10 as the top status). |
|  | Income, occupation position compared with the relatives, friends and colleagues at the current residence |  |
|  | Income, occupation position compared with friends and colleagues in their hometowns |  |
|  | Degree of respect compared with whole society(1-10) |  |
|  | Degree of respect compared with relatives, friends and colleagues of the current residence(1-10) |  |
|  | Degree of respect compared with friends and colleagues of their hometown(1-10) |  |
| Social communication | Number of   organizations participated | The number of organizations the subjects participated in, such as labor union, volunteer association, the Chinese Communist Party group of migrants/local residents, alumni association, chamber of commerce of hometown, association of migrants from the same hometown and other organizations |
|  | Number of activities attended | The number of activities the subjects participated in, such as community sports, social public welfare activities, election campaign, awards events, the home owners' committee, management activities of residents' committees and other activities |
|  | Type of neighbors | Whether the neighbors of the subjects were registered residents, who had “HuKou”, or migrants. |
| Acculturation | The consent of the views | Those views include 7 items about social norms: 1) The customs at hometown (such as the customs of marriage, funerals) is more important to the subject; 2) Working in the current place is more important to me than living at hometown; 3) The subject’s child should learn to speak hometown dialect; 4) Maintaining the hometown's lifestyle, such as eating habits, is important; 5) There is a big difference on health habits between the subject and local residents; 6) There is a big difference on clothing between the subject and local residents; 6) There is a big difference on education or retirement style between the subject and local residents; 7) The subject’s opinions of some social problems are very different from the local residents’. Respondents were asked to report their agreements with these views based on five point scale (strongly agree, agree, neither agree or not, disagree, strongly disagree) |
| Self-identity | Integration will | Integration will consists of 13 questions, such as “I would like to live together with locals in a block (community)”, “I would like to be a colleague with locals” and “I would like to be neighbor with locals”. Respondents were asked about the level of agreement with these statements based on four point scales (1 as disagree completely and 4 as agree completely) and the higher score means better integration will |
|  | Willingness to bring family members to local residence | Whether to bring the subject's spouse, unmarried children, or parents to local residence in the next 1 to 3 years. |
